# Quercetin‐Driven *Akkermansia Muciniphila* Alleviates Obesity by Modulating Bile Acid Metabolism via an ILA/m^6^A/CYP8B1 Signaling

**DOI:** 10.1002/advs.202412865

**Published:** 2025-01-31

**Authors:** Jiaqi Liu, Youhua Liu, Chaoqun Huang, Chuan He, Tongyudan Yang, Ruiti Ren, Zimeng Xin, Xinxia Wang

**Affiliations:** ^1^ College of Animal Sciences Zhejiang University Hangzhou 310058 China; ^2^ Key Laboratory of Molecular Animal Nutrition (Zhejiang University) Ministry of Education Hangzhou 3100058 China; ^3^ Key Laboratory of Animal Nutrition and Feed Science (Eastern of China) Ministry of Agriculture and Rural Affairs Hangzhou 310058 China; ^4^ Zhejiang Key Laboratory of Nutrition and Breeding for High‐quality Animal Products Hangzhou 310058 China; ^5^ Department of Chemistry Department of Biochemistry and Molecular Biology Institute for Biophysical Dynamics Howard Hughes Medical Institute The University of Chicago 929 East 57th Street Chicago IL 60637 USA

**Keywords:** *Akkermansia muciniphila*, cholic acid, m^6^A, obesity, quercetin

## Abstract

Global health is increasingly challenged by the growing prevalence of obesity and its associated complications. Quercetin, one of the most important dietary flavonoids, is being explored as an effective therapy for obesity with its mechanism remains understudied. Here in this study, it is demonstrated that quercetin intervention significantly reverses obesity‐related phenotypes through reshaping the overall structure of microbiota, especially boosting colonization of the beneficial gut commensal *Akkermansia muciniphila* (*A. muciniphila*). Enrichment of *A. muciniphila* leads to generate more indole‐3‐lactic acid (ILA) to upregulate the expression of 12α‐hydroxylase (CYP8B1) via fat mass and obesity‐associated protein (FTO)/ *N* ^6^‐methyladenosine (m^6^A)/YTHDF2 manner, thereby facilitating cholesterol converts to cholic acid (CA). CA in turn drastically suppresses lipid accumulation via activating the farnesoid X receptor (FXR) in adipose tissue. This work introduces a novel therapeutic target for addressing obesity and expands upon the current limited understanding of the mediator function of m^6^A modifications in microorganism‐influenced bile acid (BA) metabolism.

## Graphical abstract

Quercetin intervention reshapes the overall structure of microbiota and significantly increases the abundance of beneficial gut commensal *A. muciniphila*. Enrichment of *A. muciniphila* leads to generate more ILA to facilitate cholesterol converts to CA in FTO/m^6^A/CYP8B1 coordinated manner, thereby reversing obesity‐related phenotypes via activating FXR in adipose tissue.

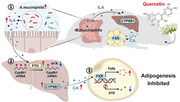

## 1 Introduction

Obesity and associated chronic metabolic diseases, which have rapidly prevailed worldwide over the past several decades, have emerged as a substantial public health challenge.^[^ ^1^, ^2^ ^]^ Growing interest has been sparked in developing effective therapeutic strategies to ameliorate obesity and metabolic dysregulation. As an alternative to pharmacological approaches, natural plant polyphenols are being explored as a potentially effective therapy for obesity due to their high efficacy and minimal risk of side effects, despite the poorly understood underlying mechanisms.

Quercetin (3,3′,4′,5,7‐pentahydroxy‐2‐phenylchromen‐4‐one),^[^ ^3^ ^]^ a representative natural flavonoid widely abundant in various types of herbs and food, holds particular promise as a treatment for a variety of diseases due to its antioxidant, anti‐obesity, anti‐aging, anti‐inflammatory, antiviral, and anti‐neoplastic properties.^[^ ^4^, ^5^, ^6^ ^]^ Previous studies have revealed that protective action of quercetin involves NLR family pyrin domain containing 3 (NLRP3) inflammasome,^[^ ^7^ ^]^ nuclear factor kappa B (NF‐κB),^[^ ^8^ ^]^ sirtuin 1 (SIRT1)/Akt pathway,^[^ ^9^ ^]^ mitogen‐activated protein kinase (MAPK), c‐Jun N‐terminal kinase (JNK), extracellular signal‐regulated kinase (ERK) and many other anti‐inflammatory pathways.^[^ ^5^, ^7^, ^10^ ^]^ It is noteworthy that gut microbiota serves as an important intermediary in the regulation of dietary nutrition on host biological processes. Quercetin commonly and indirectly exerts its probiotic functions through interacting with gut microbiota owing to the complex chemical structure features.^[^ ^4^, ^11^ ^]^ Porra et al. discovered that quercetin mitigates nonalcoholic fatty liver disease (NAFLD) by modulating intestinal microbiota imbalance.^[^ ^12^ ^]^ Trying to explore from the perspective of microorganisms and then identify precise functions of specific bacteria can help to improve mechanistically understand of quercetin.

*N* ^6^‐methyladenosine (m^6^A) is the most prevalent RNA modification in eukaryotes and acts as a critical player in fine‐tuning mRNA metabolism, including mRNA splicing, export, localization, translation, and stability.^[^ ^13^, ^14^ ^]^ Extensive evidence has shown a strong connection between m^6^A modification of mRNA and the pathogenesis of various diseases, especially obesity.^[^ ^1^ ^]^ Notably, *Akkermansia muciniphila* (*A. muciniphila*) and *Lactobacillus plantarum* alters specific m^6^A modifications in cecum and liver.^[^ ^15^ ^]^ *Fusobacterium nucleatum* induces a dramatic decline in METTL3‐mediated m^6^A modifications in colorectal cancer (CRC) cells, exacerbating CRC aggressiveness.^[^ ^16^ ^]^ Heat‐killed *Salmonella typhimurium* infection markedly raises the global m^6^A levels of mRNA in tohoku hospital pediatrics‐1 (THP‐1) cells.^[^ ^17^ ^]^ All these studies imply that m^6^A methylation may exert an unignored role in the modulation of dietary nutrition or gut microbiota on host biological processes.

In this study, we screened and identified *A. muciniphila* as a crucial mediator in dietary quercetin‐caused metabolic dysregulation improvements. Mechanistically, we demonstrated that *A. muciniphila* and its metabolite indole‐3‐lactic acid (ILA) modulate cholic acid (CA) metabolism by increasing 12α‐hydroxylase (CYP8B1) expression in FTO‐m^6^A‐YTHDF2 signaling. Upregulated expression of CYP8B1 facilitates the conversion of cholesterol to CA, which in turn significantly suppresses lipid accumulation via activating the farnesoid X receptor (FXR) in adipose tissue.

## 2 Results

### 2.1 Quercetin Ameliorates HFD‐Induced Obesity and Related Metabolic Dysregulation

**Figure 1:**
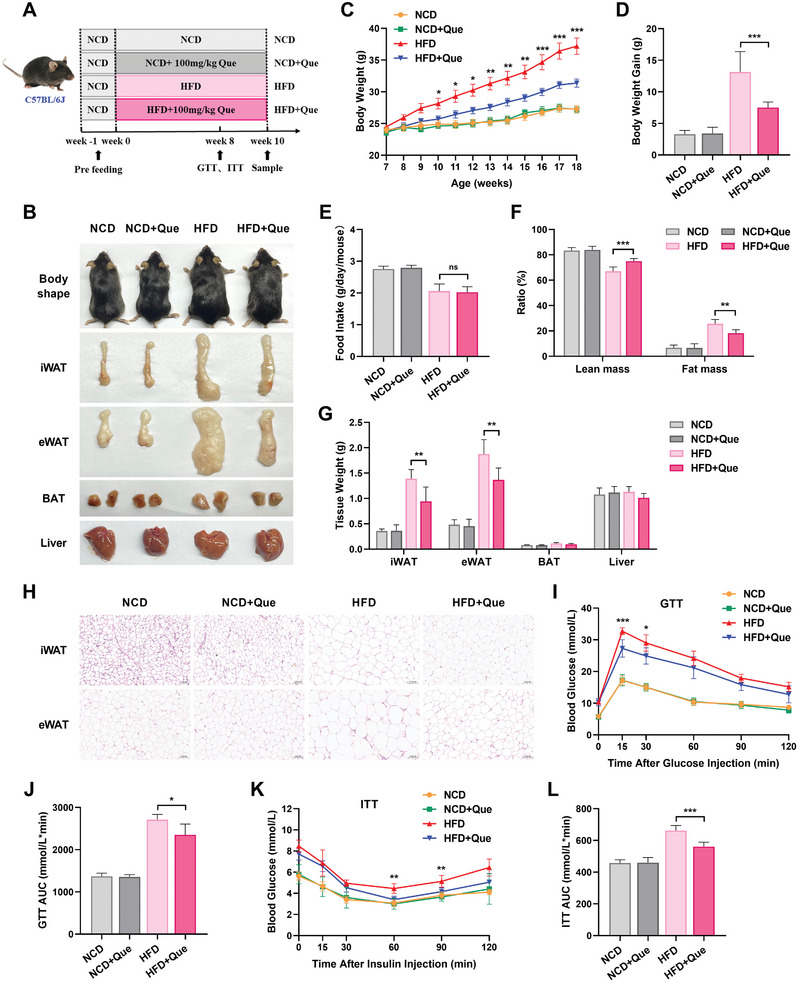
Quercetin ameliorates diet‐induced adiposity and associated metabolic disorders. A) Schematic illustration of the experimental design for administering quercetin to C57BL/6J mice. B) Representative photographs of mice body shape, iWAT, eWAT, BAT, and liver. C) Dynamic changes in body weight of mice (*n *= 9). D) Relative body weight gain of mice at termination of study (*n *= 9). E) Food intake of mice. F) Body composition parameters of lean and fat tissues of mice (*n *= 6). G) iWAT, eWAT, and BAT weights at termination of study (*n *= 6). H) Representative H&E staining of iWAT and eWAT from mice. Scale bars, 100 µm. I,J) Blood glucose levels of mice after intraperitoneal injection of glucose for glucose tolerance tests (GTT). The area under the curve (AUC) was calculated based on GTT results (I) (*n *= 6). K,L) Blood glucose levels of mice after intraperitoneal injection of insulin for insulin tolerance tests (ITT). The area under the curve (AUC) was calculated based on ITT results (L) (*n *= 6).

To investigate the physiological impact of quercetin on metabolism and fat accumulation in vivo, male C57BL/6J mice were subjected to normal chow diet (NCD), NCD supplemented with quercetin (NCD+Que), high‐fat diet (HFD), or HFD supplemented with quercetin (HFD+Que) for a duration of 10 weeks (**Figure** **1**A). As the representative photographs showed in Figure 1B, HFD mice developed typical obesity characteristics compared with NCD mice, indicating the successful establishment of the obesity model. Oral administration of quercetin led to a significant reduction in body weight (Figure 1B–D), fat mass percentage (Figure 1F), inguinal white adipose tissue (iWAT), and epididymal WAT (eWAT) (Figure 1B,G) in mice under high‐fat conditions, despite comparable daily average food intake (Figure 1E). Moreover, quercetin gavage could effectively mitigate the enlargement of adipocytes in WAT caused by HFD, as evidenced by hematoxylin and eosin (HE) staining (Figure 1H). Given that obesity is commonly associated with metabolic irregularities, we also employed glucose tolerance test (GTT) and insulin tolerance test (ITT) to assess the role of quercetin in maintaining metabolic homeostasis. As anticipated, HFD+Que mice exhibited improved glucose disposal ability (Figure 1I,J) and increased insulin sensitivity (Figure 1K,L) compared with HFD mice. Nevertheless, under NCD‐feeding, no significant variations were observed in food consumption, body weight, body composition, tissue weight, adipocyte size, glucose tolerance, or insulin sensitivity following quercetin treatment in mice (Figure 1). In summary, these observations demonstrate that quercetin offers protection against HFD‐induced obesity and related metabolic dysfunctions.

### 2.2 Quercetin Reshapes the Overall Structure of Microbiota, Specially Elevates *A. Muciniphila* Abundance

**Figure 2:**
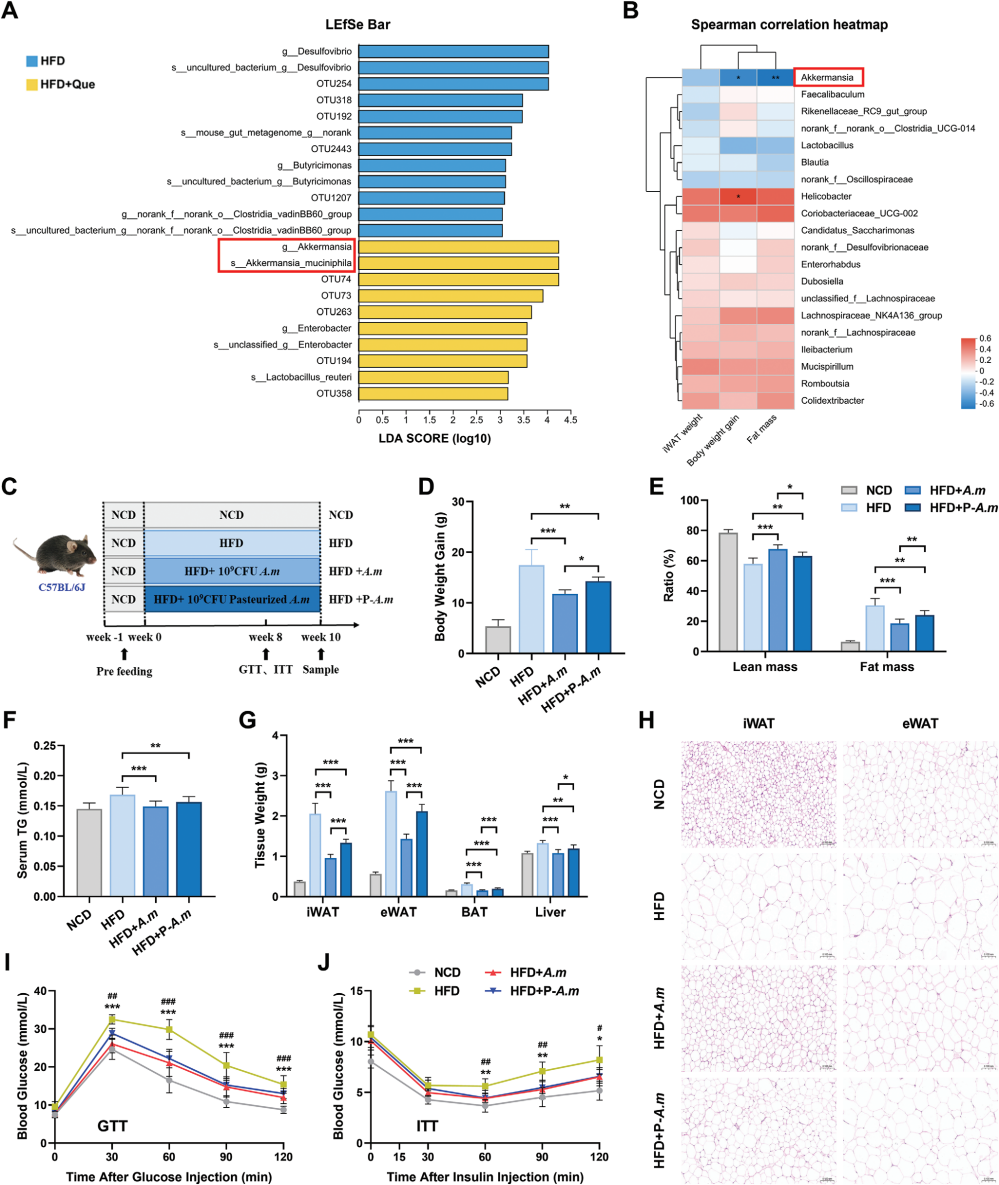
Quercetin restructures the microbiome and increases *A. muciniphila* abundance. A) LEfSe analysis identifies essential differences in bacterial abundance (Genus to OUT level) between HFD and HFD+Que groups. Only taxa with LDA score greater than 3 are shown (*n *= 9). B) Spearman correlation coefficients between fecal bacterial abundance and selected metabolic parameters (NCD *n *= 7, NCD+Que *n *= 7, HFD *n *= 9, HFD+Que *n *= 9). C) Schematic illustration of the experimental design for administering *A. muciniphila* to C57BL/6J mice. D) Relative body weight gain of mice at termination of study (*n *= 8). E) Body composition parameters of lean and fat tissues of mice (*n *= 8). F) Serum levels of total triglyceride (TG) in mice (*n *= 8). G) iWAT, eWAT and BAT weights at termination of study (*n *= 8). H) Representative H&E staining of iWAT and eWAT from mice. Scale bars, 100 µm. I) Blood glucose levels of mice after intraperitoneal injection of glucose for GTT (*n *= 8). ^*^ significant difference between HFD and HFD+*A. m* groups. ^#^ significant difference between HFD and HFD+P‐*A. m* groups. J) Blood glucose levels of mice after intraperitoneal injection of insulin for ITT (*n *= 8). ^*^ significant difference between HFD and HFD+*A. m* groups. ^#^ significant difference between HFD and HFD+P‐*A. m* groups.

Quercetin, a naturally occurring polyphenol, is generally poorly bioavailable because of its intricate chemical structure and polymeric forms.^[^ ^4^, ^6^ ^]^ Up to 90%–95% of dietary polyphenols cannot be absorbed in the small intestine but instead pass to the colon, where they interact with gut microbiota to exert their probiotic functions.^[^ ^11^ ^]^ Considering this, we subsequently analyzed the gut microbiota configurations modified by quercetin through 16S rDNA amplicon sequencing. Hierarchical clustering tree outcomes (Figure S1A, Supporting Information) and principal coordinate analysis (PCoA) based on Bray‐Curtis distance (Figure S1B, Supporting Information) at the OTU level both showed a significant separation in microbiota structure among the four groups. HFD mice displayed substantially reduced microbial diversity (Shannon index) (Figure S1C, Supporting Information) and microbial richness (Ace index) (Figure S1D, Supporting Information) compared with NCD mice, while quercetin gavage mitigated the reduction and exhibited a higher gut microbiome health index (GMHI) upon HFD‐feeding (Figure S1E, Supporting Information). What's more, the relative abundance of many bacteria was markedly altered between HFD and HFD+Que mice (Figure S1F–H, Supporting Information). To further screen out the key differential bacterial genera, we performed LEfSe analysis and Wilcoxon rank‐sum test. Notably, genus *A. muciniphila*, a promising probiotic,^[^ ^18^ ^]^ was enriched in HFD+Que mice (**Figure** **2**A; Figure S1I, Supporting Information). Its relative abundance was also higher due to quercetin treatment under basal diet conditions (Figure S1J, Supporting Information). Besides, Spearman's correlation analysis revealed that *A. muciniphila* was the only microorganism dramatically and inversely correlated with metabolic parameters, such as body weight gain, fat mass percentage, and weight of iWAT (Figure 2B). The sequencing results above indicate that quercetin supplementation alters microbiota structure and increases *A. muciniphila* abundance, leading us to hypothesize that *A. muciniphila* may play a crucial role in quercetin‐induced metabolic improvements.

To determine whether *A. muciniphila* could alleviate metabolic dysregulation and to define the protective component of *A. muciniphila*, we orally administered either phosphate‐buffered saline (PBS), viable *A. muciniphila*, or pasteurized *A. muciniphila* to male C57BL/6J mice under HFD‐feeding for a duration of 10 weeks, referred to as HFD, HFD+ *A. m*, and HFD+P‐*A. m*, respectively (Figure 2C). The obesity model was successfully constructed (Figure S2A, Supporting Information). *A. muciniphila* treatment effectively increases the abundance of *A. muciniphila* in mice feces (Figure S2B, Supporting Information). Consistent with the outcomes of quercetin treatment (Figure 1) and previous literatures,^[^ ^18^, ^19^, ^20^ ^]^ no significant alteration in daily average food consumption was observed after viable or pasteurized *A. muciniphila* treatment (Figure S2D, Supporting Information), while their supplementation resulted in lower body weight (Figure 2D; Figure S2C, Supporting Information) and fat mass percentage (Figure 2E) compared with PBS‐treated HFD mice, predominantly owing to the significant compromise in iWAT, eWAT, and brown adipose tissue (BAT) depots (Figure 2G). What's more, a remarkable decrease in serum total triglyceride (TG) (Figure 2F) and adipocyte size (Figure 2H), as well as an improvement in glucose homeostasis (Figure 2I; Figure S2E, Supporting Information) and insulin sensitivity (Figure 2J; Figure S2F, Supporting Information), were also noted in HFD+ *A. m* and HFD+P‐*A. m* mice. Although both viable and pasteurized *A. muciniphila* demonstrated potential in addressing obesity, it is important to highlight that viable *A. muciniphila* exhibited more pronounced protective properties, suggesting that the observed protective effect is likely primarily dependent on microbial metabolites rather than the bacterial components. Taken together, these findings confirm that *A. muciniphila* is beneficial for metabolic homeostasis and is negatively correlated with obesity characteristics.

### 2.3 *A. Muciniphila* Increases Serum CA Levels, Which Suppresses Lipid Accumulation via FXR Signaling

**Figure 3:**
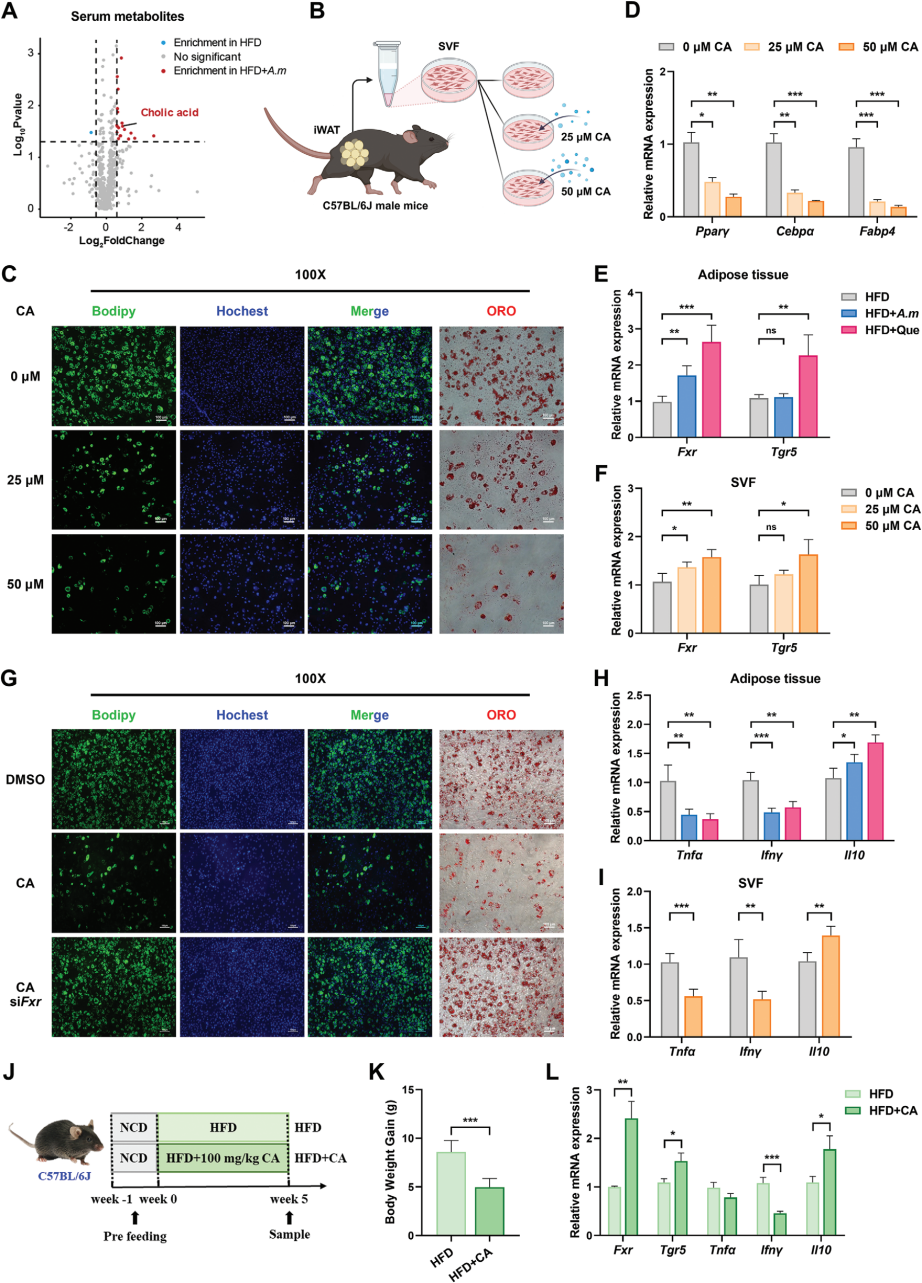
*A. muciniphila*‐driven CA confers protection against obesity via FXR signaling. A) Volcano plot showing differentially expressed serum metabolites induced by *A. muciniphila* colonization. The indicate plot was CA (*n *= 6). B) Schematic of cellular experiments in vitro. The primary stromal vascular fraction (SVF) cells were isolated from iWAT of male C57BL/6J mice, induced to differentiate, and treated with 0, 25, or 50 µm of CA throughout the differentiation process. C) Bodipy and ORO staining of SVF cells. Differentiation was induced with CA up to day 6. D) mRNA expression levels of adipogenesis‐related marker genes in SVF cells (*n *= 4). E,F) mRNA expression levels of major BAs receptors (*Fxr* and *Tgr5*) in iWAT (E) and SVF cells (F) (*n *= 4). G) Bodipy and ORO staining of SVF cells in *Fxr*‐silenced or CA‐treated groups. H,I) mRNA expression levels of *Tnfα*, *Ifnγ*, and *Il10* in iWAT (H) and SVF cells (I) (*n *= 4). J) Schematic illustration of the experimental design for administering CA to C57BL/6J mice. K) Relative body weight gain of mice at termination of study (*n *= 6). L) mRNA expression levels of *Fxr*, *Tgr5*, *Tnfα*, *Ifnγ*, and *Il10* in iWAT (*n *= 3).

Metabolites produced or influenced by microorganisms bridge the dynamic cross‐talk between microbiotas and peripheral target tissues.^[^ ^21^ ^]^ To identify potential metabolites that could regulate lipid deposition, untargeted metabolomics profiling of the serum samples collected from HFD and HFD+*A. m* mice were conducted. Partial least squares‐discriminant analysis (PLS‐DA) indicated a substantially altered circulating metabolites composition after *A. muciniphila* gavage (Figure S3A, Supporting Information). A collective of 20 metabolites exhibited notable alterations: 19 metabolites were distinctly upregulated, while 1 metabolite was markedly downregulated in HFD+*A. m* mice in comparison with HFD mice (**Figure** **3**A; Figure S3B, Supporting Information). These differential metabolites were enriched in metabolic pathways, primary bile biosynthesis, bile secretion, vitamin B6 metabolism, tyrosine metabolism, and some other biological processes (Figure S3C, Supporting Information). Among them, we particularly focus on cholic acid (CA), one of the primary BAs, as it was enriched in multiple pathways (Figure S3D,E, Supporting Information) and many studies reported that BAs have important regulatory effects against metabolic, inflammatory, infectious, and neoplastic diseases.^[^ ^22^, ^23^ ^]^ Intriguingly, the circulating CA levels were also predominantly elevated following quercetin supplementation under HFD‐feeding (Figure S3F, Supporting Information). Based on above results, we speculated that CA driven by *A. muciniphila* might suppress lipid accumulation. To validate this, primary stromal vascular fraction (SVF) cells were isolated from iWAT of male C57BL/6J mice, induced to differentiate in vitro, and treated with various concentrations of CA throughout the differentiation process (Figure 3B). As expected, adipogenic differentiation was strikingly inhibited by CA in a concentration‐dependent manner (Figure 3C; Figure S3G, Supporting Information), while CA treatment did not cause any notable impact on cell viability (Figure S3I, Supporting Information). The mRNA expression of adipogenesis master regulators, including *Pparγ*, *Cebpα*, and *Fabp4*, was all attenuated by CA in a dose‐dependent manner as well (Figure 3D). Except for CA, alpha‐linolenic acid and additional 18 metabolites may also possess anti‐obesity properties. Other potential metabolites or biological pathways could be an important direction for future exploration.

How does CA confer anti‐obesity responses? Numerous studies revealed that BAs regulate symbiotic metabolic networks by binding and activating different receptors, particularly farnesoid X receptor (FXR) and Takeda G‐protein‐coupled receptor 5 (TGR5).^[^ ^23^, ^24^ ^]^ CA has been confirmed to have FXR/TGR5‐activating potential. In our current study, we observed substantially activation of FXR in iWAT following *A. muciniphila* or quercetin supplementation, compared with mice solely on HFD regimen (Figure 3E). Similarly, only FXR was markedly activated when treated SVF cells with various concentrations of CA in vitro (Figure 3F). To further verify the role of FXR, we carried out siRNA‐mediated rescue experiments and discovered that depleting FXR could partially reverse the inhibition of lipid deposition induced by CA, implicating that CA exerts its function indeed requires FXR (Figure 3G; Figure S3H, Supporting Information). Since FXR is a transcription factor for inflammation‐related genes and inflammation is a key component of obesity, we next detected the expression of marker genes. Data showed that *A. muciniphila* or quercetin gavage led to upregulation of anti‐inflammatory factors (*Il10*) and downregulation of pro‐inflammatory factors (*Tnfα* and *Ifnγ*) in iWAT (Figure 3H). Analogous changes were seen in CA‐treated SVF cells (Figure 3I). Furthermore, we orally administered either PBS or CA to mice under HFD‐feeding to investigate the in vivo anti‐obesity effects of CA (Figure 3J). Not surprisingly, CA exhibited a considerable ability to mitigate obesity (Figure 3K; Figure S3J–M, Supporting Information), activate FXR, and modulate the expression of inflammation‐related genes (Figure 3L). Together, our data unveil that *A. muciniphila* could modulate BA metabolism. Increased circulating CA levels suppressed lipid accumulation in a manner dependent on the BAs receptor FXR.

### 2.4 *A. Muciniphila* Metabolites Enhance CYP8B1 Expression in an m^6^A‐Dependent Manner, Thereby Upregulating CA Levels

**Figure 4:**
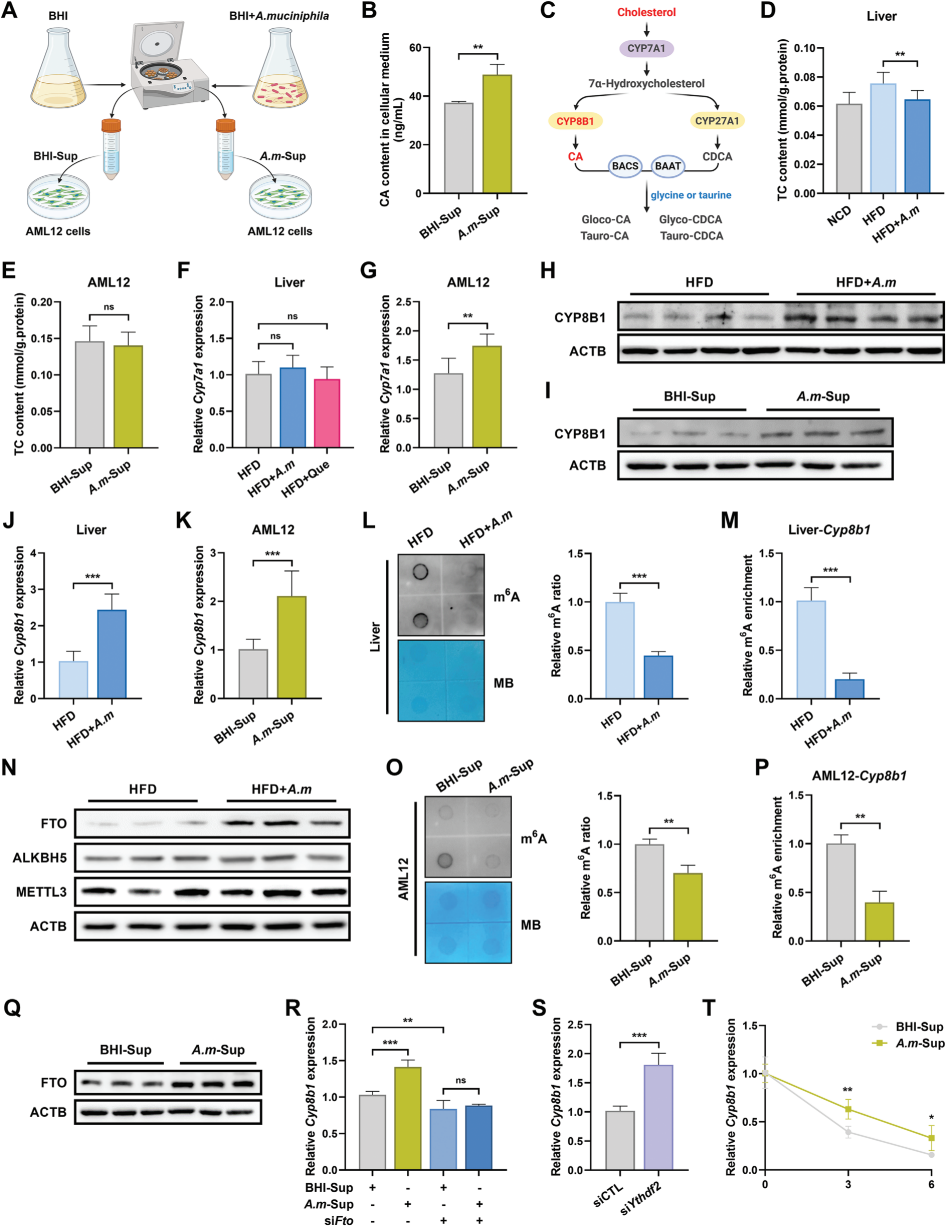
*A. muciniphila* metabolites enhances CYP8B1 expression in an m^6^A‐dependent manner to generate more CA. A) Experimental schematic for collecting *A. muciniphila* cultural supernatants to treat AML12 cells, with blank BHI supernatants as control. B) CA concentrations in AML12 cells cultural medium (*n *= 3). C) Schematic of the BAs metabolism pathway. D) Total cholesterol (TC) content of livers in mice (*n *= 8). E) TC content of AML12 cells treated with BHI‐Sup or *A. m*‐Sup (*n *= 4). F,G) mRNA expression levels of *Cyp7a1* in livers (F) (*n *= 6) and AML12 cells (G) (*n *= 6). H,I) Protein expression levels of CYP8B1 in livers (H) (*n* = 4) and AML12 cells (I) (*n *= 3). J,K) mRNA expression levels of *Cyp8b1* in livers (J) (*n *= 8) and AML12 cells (K) (*n *= 6). L) mRNA m^6^A modification levels in livers of mice. Methylene blue staining was used as a loading control (*n *= 3). M) m^6^A modification levels of *Cyp8b1* mRNA in livers of mice (*n *= 3). N) Protein expression levels of m^6^A regulator in livers of mice (*n *= 3). O) mRNA m^6^A modification levels in AML12 cells treated with BHI‐Sup or *A. m*‐Sup. Methylene blue staining was used as a loading control (BHI‐Sup *n *= 3, *A. m*‐Sup *n *= 4). P) m^6^A modification levels of *Cyp8b1* mRNA in AML12 cells treated with BHI‐Sup or *A. m*‐Sup (*n *= 3). Q) Protein expression levels of FTO in AML12 cells treated with BHI‐Sup or *A. m*‐Sup (*n *= 3). R) mRNA expression levels of *Cyp8b1* in AML12 cells were measured under different conditions: BHI‐Sup, *A. m*‐Sup, BHI‐Sup+si*Fto*, or *A. m*‐Sup+si*Fto* (*n *= 5). S) mRNA expression levels of *Cyp8b1* in *Ythdf2* siRNA or negative siRNA‐transfected AML12 cells (*n *= 7). T) Lifetime of *Cyp8b1* mRNA in AML‐12 cells treated with BHI‐Sup or *A. m*‐Sup (*n *= 4).

BAs are primarily synthesized in the classic pathway by hepatocytes.^[^ ^22^ ^]^ To further identify if *A. muciniphila* also influences CA levels in vitro, we added *A. muciniphila* cultural supernatants (*A. m*‐Sup) into the AML12 cells medium, with blank brain heart infusion (BHI) medium supernatants (BHI‐Sup) as control (**Figure** **4**A). In line with observations in vivo, CA content in hepatocytes cultural medium was strikingly elevated due to *A. m*‐Sup treatment (Figure 4B). How *A. muciniphila* metabolites induce an increase CA biosynthesis is the next question we aim to decipher.

According to prior literature, cholesterol is metabolized to 7α‐hydroxycholesterol by the rate‐limiting enzyme cholesterol 7α‐hydroxylase (CYP7A1), and then converted to CA or chenodeoxycholic acid (CDCA) by sterol 12α‐hydroxylase (CYP8B1) or sterol 27‐hydroxylase (CYP27A1), respectively (Figure 4C).^[^ ^22^, ^25^ ^]^ Here, we first excluded the possibility that *A. muciniphila* colonization produced more cholesterol to generate more CA. This was demonstrated through lower hepatic concentrations of total cholesterol (TC) in HFD+ *A. m* mice (Figure 4D), as well as unchanged TC levels in hepatocytes following *A. m*‐Sup treatment (Figure 4E). The expression of *Cyp7a1* could be effectively upregulated by *A. m*‐Sup in vitro (Figure 4G); nonetheless, it was not significantly altered by quercetin or *A. muciniphila* intervention in vivo (Figure 4F). Regarding the expression of CYP8B1, livers of HFD+ *A. m* mice (Figure 4H,J; Figure S4A, Supporting Information), livers of HFD+Que mice (Figure S4C,D, Supporting Information), and hepatocytes treated with *A. m*‐Sup (Figure 4I,K; Figure S4B, Supporting Information) showed a significant increase compared with control group. All these results suggest that *A. muciniphila* metabolites augment serum CA levels via enhancing CYP8B1 expression. However, the regulatory mechanism governing the upregulation of CYP8B1 expression requires further clarification.

Jabs et al. proposed that germ‐free (GF) mice mono‐colonized with *A. muciniphila* displayed a notable alteration on m^6^A modifications in the livers.^[^ ^15^ ^]^ RNA m^6^A modification plays a crucial role in host metabolic health.^[^ ^1^, ^26^ ^]^ What’ more, 24 m^6^A sites of *Cyp8b1* mRNA have been recorded in RMBase v3.0 (http://bioinformaticsscience.cn/rmbase/).^[^ ^27^ ^]^ These factors prompted us to verify if the augmentation of CYP8B1 expression depends on m^6^A modifications. Coincides with our hypothesis, hepatic overall m^6^A levels are lower in HFD+ *A. m* mice than in HFD mice (Figure 4L). The levels of m^6^A‐modified *Cyp8b1* mRNA were also decreased owing to *A. muciniphila* supplementation (Figure 4M). Since the abundance of m^6^A on mRNA is determined by the dynamic interplay between major methyltransferases (METTL3 and METTL14) and demethylases (FTO and ALKBH5), we next examined and found that mitigated m^6^A levels attributed to enhanced FTO expression (Figure 4N; Figure S4F, Supporting Information). Livers of HFD+Que mice exhibited decreased m^6^A levels (Figure S4E, Supporting Information) and increased FTO protein expression (Figure S4C,D, Supporting Information) as well. Identically, treating AML12 cells with *A. m*‐Sup yielded same outcomes, including lower total m^6^A levels (Figure 4O), reduced levels of m^6^A in *Cyp8b1* mRNA (Figure 4P), and accelerated protein expression of FTO (Figure 4Q; Figure S4G, Supporting Information). Besides, the further loss‐of‐function studies ascertained the mediator role of FTO, manifested as perturbing FTO indeed offset the impact of *A. m*‐Sup on *Cyp8b1* mRNA levels (Figure 4R).

m^6^A modifications exert an effect on mRNA primarily by recruiting RNA‐binding proteins (T521‐B homology (YTH) domain‐containing proteins), also known as “m^6^A reader.”^[^ ^28^ ^]^ YTHDF2, one of the m^6^A readers, has been documented to selectively recognize m^6^A sites and mediate m^6^A‐containing mRNA degradation. Knockdown of *Ythdf2* caused higher mRNA expression of *Cyp8b1* (Figure 4S), implying that YTHDF2 is involved in *Cyp8b1* transcription. The m^6^A‐containing *Cyp8b1* mRNA may be recognized and bound by YTHDF2 and then degraded. To test this, we detected the relative mRNA content of *Cyp8b1* at 0, 3, and 6 h after adding actinomycin D to block transcription. Data showed that *A. m*‐Sup distinctly extended the half‐life of *Cyp8b1* mRNA (Figure 4T). Collectively, we discover for the first time that *A. muciniphila* modulates BA metabolism from an epigenetics perspective, thereby alleviating host obesity.

### 2.5 The Metabolite ILA is Responsible for the Increased m^6^A‐Mediated CYP8B1 Expression

**Figure 5:**
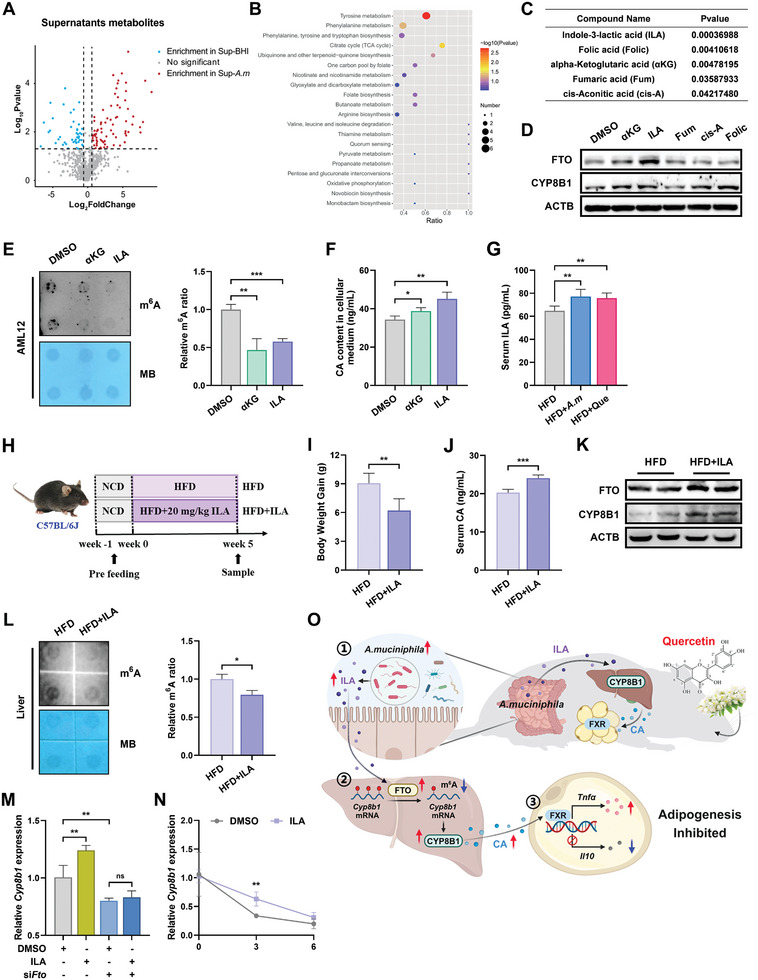
The metabolite ILA is accountable for the upregulated m^6^A‐mediated CYP8B1 levels. A) Volcano plot showing differentially expressed metabolites between blank BHI supernatants and *A. muciniphila* cultural supernatants (*n * = 3). B) KEGG enrichment analysis of differentially expressed metabolites described in (A) (*n *= 3). C) The list of screened potential metabolites derived from *A. muciniphila*. D) Protein expression levels of FTO and CYP8B1 in AML12 cells treated with metabolites listed in (C) (*n *= 3). E) mRNA m^6^A modification levels in AML12 cells treated with DMSO, αKG, or ILA (*n *= 3). F) CA concentrations in AML12 cells cultural medium (*n *= 3). G) Serum levels of ILA in mice between HFD, HFD+*A.m*, and HFD+Que groups (*n *= 6). H) Schematic illustration of the experimental design for administering ILA to C57BL/6J mice. I) Relative body weight gain of mice at termination of study (*n *= 6). J) Serum levels of CA in mice between HFD and HFD+ILA groups (*n *= 4). K) Protein expression levels of FTO and CYP8B1 in livers of mice from HFD and HFD+ILA groups (*n *= 3). L) mRNA m^6^A modification levels in livers of mice from HFD and HFD+ILA groups (*n *= 3). M) mRNA expression levels of *Cyp8b1* in AML12 cells were measured under different conditions: DMSO, ILA, DMSO+si*Fto*, or ILA+si*Fto* (DMSO *n *= 4, ILA *n *= 4, DMSO+si*Fto n* = 4, ILA+si*Fto n *= 3). N) Lifetime of *Cyp8b1* mRNA in AML‐12 cells treated with DMO or ILA (*n *= 4). O) Working model of quercetin/*A. muciniphila* in regulating fat accumulation.

Subsequently, we examined the *A. muciniphila* cultural supernatants via untargeted metabolome analyses and identified 77 metabolites produced by *A. muciniphila* (**Figure** **5**A). The possibility of *A. muciniphila* directly generates more CA was ruled out (Figure S5A, Supporting Information). KEGG analysis showed marked enrichment in tyrosine metabolism, phenylalanine metabolism, citrate cycle (TCA cycle), one carbon pool by folate, and other pathways (Figure 5B). Indole‐3‐lactic acid (ILA), folic acid (Folic), alpha‐ketoglutaric acid (αKG), fumaric acid (Fum), and cis‐aconitic acid (cis‐A) were speculated as potential candidates for additional investigation based on their previously reported functions (Figure 5C). When utilizing these aforementioned metabolites on AML12 cells, two of them, ILA and αKG, appeared to exhibit higher protein levels of FTO and CYP8B1 (Figure 5D; Figure S5B,C, Supporting Information), lower cellular m^6^A levels (Figure 5E), as well as higher CA concentrations (Figure 5F) compared with control group, with the most significant effects observed in ILA‐treated hepatocytes. What's more, serum ILA levels were predominantly elevated following *A. muciniphila* and quercetin supplementation (Figure 5G). To validate the role of ILA in vivo, mice were orally administered with either PBS or ILA (Figure 5H). As expected, ILA was able to drastically ameliorate HFD‐induced obesity (Figure 5I; Figure S5D–G, Supporting Information) without affecting food intake (Figure S5E, Supporting Information). Circulation CA levels (Figure 5J), expression of FTO and CYP8B1 (Figure 5K; Figure S5H,I, Supporting Information), total m^6^A levels in mRNA (Figure 5L), rescue effect of silenced‐FTO (Figure 5M), and lifespan of *Cyp8b1* mRNA (Figure 5N) also supported the idea that ILA contributed to m^6^A modifications‐mediated CYP8B1 upregulation. ILA has been mentioned to affect epithelium‐macrophage interaction through histone modifications.^[^ ^29^ ^]^ Our work sheds light on new insights into the epigenetic regulation of ILA.

## 3 Discussion

Gut microbiota is one of the pivotal factors closely correlated with aspects of host health, and its composition can be rapidly and substantially altered through nutrition.^[^ ^30^, ^31^, ^32^, ^33^ ^]^ With the increasing knowledge of the complicated interaction between microorganism and host, accumulating studies nowadays focus on the precise functions of specific bacteria rather than just crude correlation analysis.^[^ ^31^, ^34^ ^]^ Researches on bacterial species or strain levels are urgently required to conducted. Here, we demonstrated that dietary quercetin intervention can ameliorate obesity and associated metabolic syndrome by remodeling the overall microbiota structure, especially the strikingly increase of *A. muciniphila* abundance. *A. muciniphila*, discovered and characterized two decades ago,^[^ ^35^ ^]^ is the paradigm for next‐generation beneficial microorganisms. Perturbations of this commensal bacterium were implicated in the development of obesity and various other diseases,^[^ ^18^, ^20^, ^34^, ^36^ ^]^ while understanding of the underlying mechanism is largely limited. Our current studies described a novel *A. muciniphila*‐m^6^A‐BAs axis that suppresses host adipogenesis via activating FXR in iWAT. This work may serve as an intervention strategy for combating metabolic syndrome.

In fact, previous human and animal model‐based studies have confirmed the anti‐obesity potential of quercetin as demonstrated in this study. Administration of 100 mg day^−1^ quercetin or onion extract that contained high quercetin content in obese subjected reduced body weight and body mass index (BMI) after 12 weeks.^[^ ^37^ ^]^ HFD mice supplemented with 100 mg k^−1^g day^−1^ quercetin displayed inhibited MAPK signaling in eWAT and a 40% reduction in final body weight.^[^ ^38^ ^]^ But notably, quercetin typically exhibits systemic availability as a result of its intricate chemical structure and polymeric forms.^[^ ^4^, ^6^ ^]^ Almost all dietary quercetin cannot be absorbed but instead interacts with gut microbiota to indirectly manifest probiotic functions.^[^ ^11^ ^]^ To better describe the mechanism of quercetin, we innovatively explored from the perspective of microorganisms and appraised *A. muciniphila* as a crucial mediator in quercetin‐induced metabolic improvements. Whereas, our available findings are not yet able to clearly explain the precise reason for the high *A. muciniphila* abundance in HFD+Que mice. *A. muciniphila* was found to be an exclusive mucin‐degrading specialist present in the intestine since early life.^[^ ^18^ ^]^ Quercetin has been shown to effectively promote goblet cells to secrete more mucin.^[^ ^39^ ^]^ Drawing from the available evidence, we hypothesize that quercetin intervention drives the enrichment of *A. muciniphila* by promoting mucin secretion. Additionally, a recent study proposed an interesting point that natural selection shapes bacterial evolution in all environments.^[^ ^40^ ^]^ Researchers performed near‐daily metagenomic sampling and then confirmed the within‐host evolution and adaptation of *Bacteroides fragilis*, promoting us to speculate that “adaptive evolution” of *A. muciniphila* may be another explanation for quercetin to resist the downregulated *A. muciniphila* abundance caused by high‐fat conditions.

Defining and characterizing various microbiota metabolites could help to mechanistically understand the host‐microbiota crosstalk and to identify possible biomarkers or signatures of disease. Under diet‐induced obesity conditions, we noticed a dramatically upregulation of serum CA concentrations owing to *A. muciniphila* colonization (Figure 3A). Similar *A. muciniphila*‐driven modulation of BA metabolism was first mentioned recently in systemic viral infection mice model, with study reporting that *A. muciniphila* regulated primary BAs conjugation by specifically enhancing amino acid N‐acyltransferase (BAAT) expression.^[^ ^34^ ^]^ Different from that, our mechanistic investigations revealed that *A. muciniphila* indirectly and specifically increased CYP8B1 expression through its derived ILA, thereby promoting the cholesterol convert to CA. To date, ILA has been discovered that could downregulate glycolysis, NF‐κB, and HIF signaling pathways via the aryl hydrocarbon receptor,^[^ ^41^ ^]^ and could also transcriptionally enhance function of tumor‐infiltrating CD8 T cells by changing chromatin accessibility.^[^ ^29^ ^]^ The current study is the first demonstration representing the regulatory impact of ILA on BA metabolism, which improves the comprehension of the role of *A. muciniphila* and ILA. However, we failed to identify any counterpart genes that may accountable for ILA synthesis. The specific gene(s) involved in ILA synthesis within the genome of *A. muciniphila* warrant investigation in subsequent studies, and this should be an important direction for future attention.

Another important feature of our work is the discovery that *A. muciniphila*‐ILA modulates host BA metabolism in an m^6^A‐dependent manner. Gut microbiota, along with their metabolites, have been shown to regulate host epigenetic pathways, such as DNA methylation,^[^ ^42^, ^43^, ^44^ ^]^ RNA methylation,^[^ ^15^, ^16^, ^45^, ^46^ ^]^ or histone modification,^[^ ^29^, ^47^ ^]^ although little is known about the exact mechanism and role. Multiple existing studies, including prior research endeavors conducted by our team, have indicated that mRNA m^6^A modification plays a vital role in numerous biological events, particularly metabolic health.^[^ ^1^, ^26^, ^48^, ^49^ ^]^ More importantly, the *Cyp8b1* mRNA transcript contains m^6^A modification sites. All these factors enabled us to hypothesize whether CYP8B1‐mediated BA metabolism can be modified by mRNA m^6^A methylation. Sure enough, we indeed observed that *A. muciniphila* and ILA both mitigated m^6^A levels through elevating FTO expression. m^6^A‐containing *Cyp8b1* mRNA was recognized and bound by YTHDF2 and subsequently degraded (Figure 4S). This newly discovered regulatory mechanism involving m^6^A‐BAs has never been documented before.

In conclusion, we show that quercetin‐driven *A. muciniphila* modulates host BA metabolism in ILA/FTO/m^6^A/CYP8B1/CA coordinated manner to ameliorate diet‐induced obesity (Figure 5O). Our work presented here proposes that quercetin could serve as a candidate for use in dietary supplements to prevent obesity. More importantly, it expands upon the existing limited knowledge of the mediator function of m^6^A modifications in microorganism‐influenced BA metabolism.

## 4 Experimental Section

### Animal Ethic Statement

All animal experimental procedures were approved by the Committee on Animal Care and Use and the Committee on the Ethics of Animal Experiments of Zhejiang University (ZJU20240254) and were strictly carried out in accordance with the applicable regulations for animal experiments throughout the entire experimental period.

### Animals Study

All experimental mice were purchased from the Shanghai Model Organisms Center, Inc. (Shanghai, China) and allowed to acclimatize for 1 week. They were housed (no more than four per cage) in a pathogen‐free animal facility under a 12 h light/dark cycle, 22 ± 2 °C room temperature, and 50 ± 5% humidity with unrestricted access to food and tap water. Body weight and food intake were monitored weekly throughout the entire experimental period. After the animal sacrifice at the end of the experiment, tissues were dissected, weighed, and either soaked in formalin for later morphological analysis or immediately snap‐frozen in liquid nitrogen for further analysis. Blood samples were kept at 4 °C overnight and the serum samples were obtained by centrifugation (4 °C, 3000 rpm, and 15 min).

In quercetin supplementary experiment. A total of 40 wild‐type male C57BL/6J mice, aged 7 weeks, were randomly allocated into 4 groups as follows: normal chow diet (NCD, 10% kcal from fat, Research Diets, D12450J), NCD with gavage of 100 mg k^−1^g day^−1^ quercetin (NCD+Que, Sigma–Aldrich, Q4951),^[^ ^50^, ^51^, ^52^, ^53^, ^54^ ^]^ high fat diet (HFD, 60% kcal from fat, Research Diets, D12492), and HFD with gavage of 100 mg k^−1^g day^−1^ quercetin (HFD+Que, Sigma–Aldrich, Q4951).

In *A. muciniphila* supplementary experiment. A total of 40 wild‐type male C57BL/6J mice, aged 7 weeks, were randomly allocated into 4 groups as follows: NCD, HFD, HFD with gavage of 10^9^ CFU day^−1^ viable *A. muciniphila* (HFD+*A. m*), and HFD with gavage of 10^9^ CFU day^−1^ pasteurized *A. muciniphila* (HFD+P‐*A. m*).^[^ ^55^ ^]^ For pasteurization, *A. muciniphila* was inactivated by pasteurization for 30  min at 70 °C.^[^ ^34^ ^]^

In CA supplementary experiment. A total of 12 wild‐type male C57BL/6J mice, aged 8 weeks, were randomly allocated into 2 groups as follows: HFD and HFD with gavage of 100 mg kg^−1^ CA (Aladdin, C103690).^[^ ^56^ ^]^

In ILA supplementary experiment. A total of 12 wild‐type male C57BL/6J mice, aged 8 weeks, were randomly allocated into 2 groups as follows: HFD and HFD with gavage of 20 mg kg^−1^ ILA (Aladdin, I157602).^[^ ^29^ ^]^

### Metabolic Assays

Glucose tolerance test (GTT) and insulin tolerance test (ITT) were performed after an overnight fasting. Mice were administered intraperitoneally with a dosage of 1.75 g kg^−1^ glucose (Sigma–Aldrich, G7021) or 0.75 U kg^−1^ insulin (Sigma–Aldrich, I2643). Blood glucose levels were measured in blood samples taken from the tail vein prior to 0, 15, 30, 60, 90, and 120 min after injection by AlphaTRAK glucometers (Nanjing Yuyue Electronics, China).

### Body Composition Measurement

Prior to the experiment, mice were fasted for 6 h. Body composition parameters of lean and fat tissues of mice were measured using the MesoQMR Small Animal Body Composition Analysis and Bench‐top MRI Imaging System (NIUMAG, QMR06‐090H‐PRO).

### Morphology Analysis

For hematoxylin‐eosin (H&E) staining, tissues fixed with 4% paraformaldehyde (Servicebio, G1101) were dehydrated, embedded in paraffin wax blocks, followed by slicing and staining with H&E. Images were observed and photographed using a Leica DM 3000 microscope (LEICA).

### Bacterial Strains

*A. muciniphila* was obtained from American Type Cultural Collection (ATCC, catalogue no. BAA835) and cultured in brain heart infusion medium (BHI, Oxoid, CM1135) at 37 °C under anaerobic conditions. The concentration was quantified based on the optical density at 600 nm (OD_600_).

### Primary Mouse Preadipocytes Isolation

The isolation of stromal vascular fraction (SVF) cells from inguinal white adipose tissue (iWAT) was conducted following previously described method with minor adjustments.^[^ ^57^ ^]^ Briefly, the iWAT was dissected from 8‐week‐old male C57BL/6J mice, minced with scissors, and then digested with 1 mg mL^−1^ type II collagenase (Gibco, 17101015) at 37 °C for 45 min. Digested tissue was filtered through a cell strainer (200 µm) to remove large pieces and then centrifuged at 1000 g for 4 min. After being washed twice with PBS, the cell pellets were incubated with red blood cell lysis buffer (Beyotime Biotechnology, C3702) for 1 min, followed by centrifugation at 1000 g for 4 min. Isolated SVF cells were plated into 10 cm dishes. Floating cells were removed the next day, and fresh culture medium was added.

### Cell Culture and Differentiation

The mouse hepatocyte cell line AML12 was obtained from the American Type Culture Collection (ATCC). AML12 cells were cultured in Dulbecco's Modified Eagle Medium/Nutrient Mixture F‐12 (DMEM/F12, Gibco, 11320033) containing 10% fetal bovine serum (FBS, Gibco, 10099141), 1% insulin‐transferrin‐selenium (ITS, Gibco, 41400045), and 1% penicillin‐streptomycin (NEST Biotechnology, 211092) in a humidified atmosphere at 37 °C with 5% CO_2_. To induce differentiation, AML12 cells were treated with an induction medium containing 0.5 mmol l^−1^ oleic acid (OA, Kunchuang Biotechnology, KC005) for 24 h when the cell density reached 90%–95%.

Isolated SVF cells were cultured in DMEM/F12 (Gibco, 11320033) containing 15% FBS (Gibco, 10099141) and 1% penicillin‐streptomycin (Gibco, 15140122) in a humidified atmosphere at 37 °C with 5% CO_2_. To induce differentiation, after 2 days post‐confluence, differentiation of SVF cells was induced using an induction medium containing 1 µmol l^−1^ dexamethasone (Sigma–Aldrich, D4902), 500 µmol l^−1^ IBMX (Sigma–Aldrich, I5879), 5 µg mL^−1^ insulin (Solarbio, I8040), and 0.5 µmol l^−1^ rosiglitazone (Sigma–Aldrich, PHR2932) for 2 days, followed by a differentiation medium containing 5 µg mL^−1^ insulin. Fresh differentiation medium was replaced every 2 days until SVF cells were ready for harvest, typically around day 8.

### Cell Transfection

The small interfering RNA (siRNA) and plasmids transfection were transfected using Lipofectamine RNAiMAX (Invitrogen, 13‐778030) and Lipofectamine 2000 (Invitrogen, 11668030), respectively, according to the manufacturer's instructions. The siRNAs were ordered from GenePharma and the sequences were as follows (5′‐3′).

Negative control siRNA: UUCUCCGAACGUGUCACGUTT.

Mouse *Fto* siRNA: TTAAGGTCCACTTCATCATCGCAGG.

### Bodipy Staining

Cells were washed 3 times with PBS (Biosharp, BL302A) and then stained with 1 µg mL^−1^ Bodipy 493/503 (Invitrogen, D3922) at 37 °C in the dark for 30 min. Subsequently, cells were washed 3 times with PBS, counterstained with Hoechst 33342 (Yeasen, 40732ES03) at 37 °C in the dark for 10 min, and finally washed 3 times with PBS again. Images were observed and photographed in a darkened microscopy room using a fluorescence microscope.

### Oil Red O Staining (ORO)

Cells were washed 3 times with PBS (Biosharp, BL302A), fixed with 4% paraformaldehyde (Servicebio, G1101) at room temperature for 2 h, washed 3 times with 60% isopropanol, and then stained with a filtered Oil Red O (Sigma–Aldrich, O9755) working solution for 10 min. After rinsing with distilled water, the lipid droplets dyed red were observed and photographed using a microscope. To quantify the relative lipid accumulation, Oil Red O‐stained lipid droplets were eluted in 100% isopropanol, and the absorbance of the extract was measured at 500 nm (OD_500_).

### Biochemical Index Detection

Total cholesterol (TC) and triglycerides (TG) levels were determined with commercially available kits (Nanjing Jiancheng Institute of Biological Engineering, Nanjing, China), according to the manufacture's protocol.

### Cholic Acid (CA) Content

CA levels in serum samples and cell culture supernatants were measured using the Mouse Cholic Acid ELISA Kit (Cell Biolabs, MET5007), according to the manufacture's protocol.

### Indole‐3‐Lactic Acid (ILA) Content

ILA concentration in serum samples were measured using the Mouse ILA ELISA Kit (SINOBESTBIO, YX‐23042 M), according to the manufacture's protocol.

### Western Blot

Protein in tissues or cells were extracted using the cell total protein extraction kit (Solarbio, BC3710) and determined using the BCA protein assay kit (Novoprotein, PA002). The protein lysates were collected and immediately centrifuged at 12 000 rpm for 15 min, separated by SDS‐polyacrylamide gels, and then transferred to PVDF membrane (Millipore, IPVH00010). After blocking with 5% skim milk at room temperature for 1 h, the membrane was incubated with primary antibodies at 4 °C overnight, and subsequently incubated with HRP‐conjugated secondary antibodies (Biosharp, BL001A or BL003A) at room temperature for 1 h. The membrane was visualized using Super ECL Plus (US EVERBRIGHT, S6009L). Primary antibodies are listed in Table S1 (Supporting Information).

### Quantitative Real‐Time PCR (qPCR)

Total RNA was extracted separately from tissues or cells using FreeZol Reagent (Vazyme, R71102) and reverse transcribed into complementary DNA (cDNA) by SPARKscript II One Step RT‐PCR Kit (Shandong Sparkjade Biotechnology Co., Ltd., AG0402). qPCR analysis was performed using SYBR qPCR Master Mix (Vazyme, Q71102) with the ABI Step‐One Plus Real‐Time PCR System (Applied Biosystems). Relative expression of mRNA abundance was calculated using the 2^−ΔΔCt^ method, with β‐actin used as an internal control. All qPCR reactions were carried out in triplicate. Primer sequences are listed in Table S2 (Supporting Information).

### m^6^A Dot Blot Assays

Dot blot analysis was carried out to detect the m^6^A modification levels in mRNA from tissues or cells. Briefly, mRNA was purified from total RNA using two rounds of polyA‐tail purification with the Dynabeads mRNA DIRECT kit (Thermo Fisher Scientific, 61012) and quantified with a Qubit Fluorometer (Thermo Fisher Scientific, Q33216). 400 ng mRNA were denatured by heating at 65 °C for 5 min to disrupt secondary structures, spotted on Amersham Hybond‐N+ membrane (GE Healthcare, RPN203B), and then crosslinked with UV light (twice, 1200 J cm^−2^). After blocking with 5% skim milk at room temperature for 1 h, the membrane was incubated with anti‐m^6^A antibodies (Synaptic Systems, 202003) at 4 °C overnight, and subsequently incubated with HRP‐conjugated secondary antibodies (Biosharp, BL003A) at room temperature for 1 h. The membrane was visualized using Super ECL Plus (US EVERBRIGHT, S6009L). After exposure, the membrane was stained with a methylene blue solution to verify the consistency of mRNA loaded across different groups.

### Methylated RNA Immunoprecipitation‐qPCR (MeRIP‐qPCR) Analysis

MeRIP assay refers to the previous method with slight modifications.^[^ ^57^ ^]^ Briefly, 50 µg RNA was adjusted to 100 µl and fragmented using a BioRuptor (Diagenode) with 30 s on/off cycle for 30 cycles. One‐tenth of the fragmented mRNA was saved as input control. Other fragmented mRNA was immunoprecipitated with N^6^‐methyladenosine antibody (NEB, E1610S) coupled to Protein G Magnetic Beads (NEB, S1430) in immunoprecipitation buffer at 4 °C for 4 h, followed by elution, precipitation, and saved as IP samples. m^6^A enrichment was determined by qPCR analysis. The specific primers used were designed according to an m^6^A site predictor, SRAMP, and the sequences are as follows (5′‐3′).

*Cyp8b1*‐MeRIP‐qPCR‐Forward: CCATAAGACGCCATCCCTCC.

*Cyp8b1*‐MeRIP‐qPCR‐Reverse: GGCTCGATTCCATTGAGCAAC.

### mRNA Stability Analysis

Cell cultures were supplemented with actinomycin D (Sigma‐Aldrich, A9415) at 5 µg mL^−1^ to inhibit mRNA production. Total RNA samples were collected after incubation for 0, 3, 6, and 9 h and the mRNA expression levels were detected by qPCR analysis.^[^ ^28^ ^]^

### 16s rDNA Sequencing

Fresh feces samples were collected, immediately frozen in liquid nitrogen, and stored at −80 °C. Bacterial DNA was extracted using the QIAamp DNA Stool Mini Kit (Qiagen, 51‐504) following the manufacturer's instructions. Specific primers (338F, 5′‐ACTCCTACGGGAGGCAGCAG‐3′; 806R, 5′‐GGACTACHVGGGTWTCTAAT‐3′) were utilized to amplify the V3‐V4 region of 16S rRNA gene, and the amplification products were sequenced on the Illumina MiSeq platform at Majorbio Bio‐Pharm Technology Co. Ltd. Sequencing reads were clustered into operational taxonomic units (OTUs) with 97% sequence similarity level. Bioinformatic analysis of the gut microbiota was carried out using the Majorbio Cloud platform (https://cloud.majorbio.com). The linear discriminant analysis (LDA) effect size (LEfSe) was performed to identify the significantly abundant taxa of bacteria among different groups.

### Untargeted Metabolomics Analysis

Metabolites from serum samples and bacterial cultural supernatants were extracted with buffer (methanol: water, 4:1). The LC‐MS/MS analysis of the metabolites was performed by Thermo UHPLC‐Q Exactive HF‐X system (Thermo) at Novogene Technology Co. Ltd. Bioinformatic analysis of metabolites was carried out using the NovoMagic Cloud platform (https://magic.novogene.com).

### Statistical Analysis

Statistical analysis was performed using the SPSS software (version 22), while the GraphPad Prism (version 8.0.2) was used to visualize the data for graphing. Significance between groups was analyzed using unpaired *t*‐test or one‐way ANOVA after testing for homogeneity of variances with Levene's test. Two‐way ANOVA with Bonferroni's multiple comparisons was used for comparisons of multiple factors. All date were presented as the mean ± SD and regarded *p* < 0.05 as statistically significant. * or # *p* ＜ 0.05, ** or ## *p* ＜ 0.01, and *** or ### *p* ＜ 0.001.

## Conflict of Interest

The authors declare no conflict of interest.

## Author Contributions

X.W. and J.L. conceived the project. J.L., Y.L., C.H., T.Y., R.T., and Z.X. performed experimental work under the supervision of X.W., J.L., and Y.L. were the main contributors in the conduct of the study, data collection and analysis, data interpretation. J.L. wrote and C.H. revised the manuscript. X.W. designed the project and provided the final approval of the manuscript.

## Supporting information



Supporting Information

## Data Availability

All the datasets generated in the current study are available from the corresponding author (xinxiawang@zju.edu.cn) upon reasonable request.
