# Supplementary material for: Quercetin‐Driven Akkermansia Muciniphila Alleviates Obesity by Modulating Bile Acid Metabolism via an ILA/m6A/CYP8B1 Signaling
Source: Adv Sci (Weinh). 2025 Jan 31;12(12):2412865. doi: 10.1002/advs.202412865 (PMC11948036; doi:10.1002/advs.202412865)
Supplement: Supplementary file 1 — Supporting Information [file ADVS-12-2412865-s001.docx]

**Supplementary Figure 1**


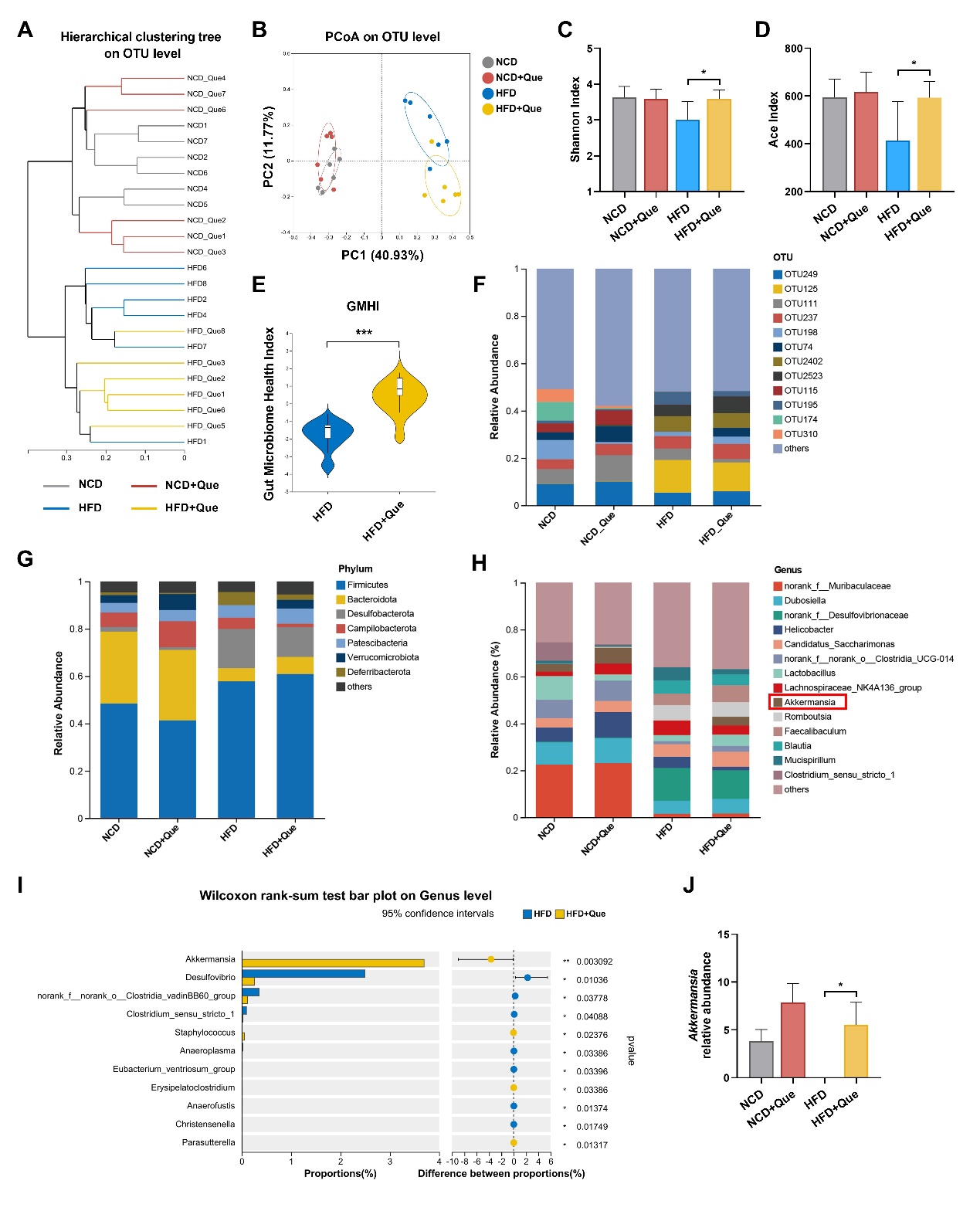


**Supplementary Figure 1. Quercetin reshapes the overall structure of microbiota**

**(A)** Hierarchical clustering tree on OUT level (*n* = 6).

**(B)** PCoA plot of β-diversity on OTU level based on Bray-Curtis distance (*n* = 6).

**(C-D)** Alpha diversity indices including Shannon **(C)** and Ace **(D)** (*n* = 6).

**(E)** Gut microbiome health index (GMHI) between HFD and HFD+Que groups (*n* = 9).

**(F-H)** Average relative proportions of bacteria at the OUT **(F)**, phylum **(G)**, and genus **(H)** levels (NCD *n* = 7, NCD+Que *n* = 7, HFD *n* = 9, HFD+Que *n* = 9).

**(I)** Bacteria with significant changes in the relative abundance in HFD and HFD+Que groups at genus level (*n* = 9).

**(J)** Relative abundance of *A. muciniphila* among the four groups (NCD *n* = 7, NCD+Que *n* = 7, HFD *n* = 9, HFD+Que *n* = 9).

**Supplementary Figure 2**


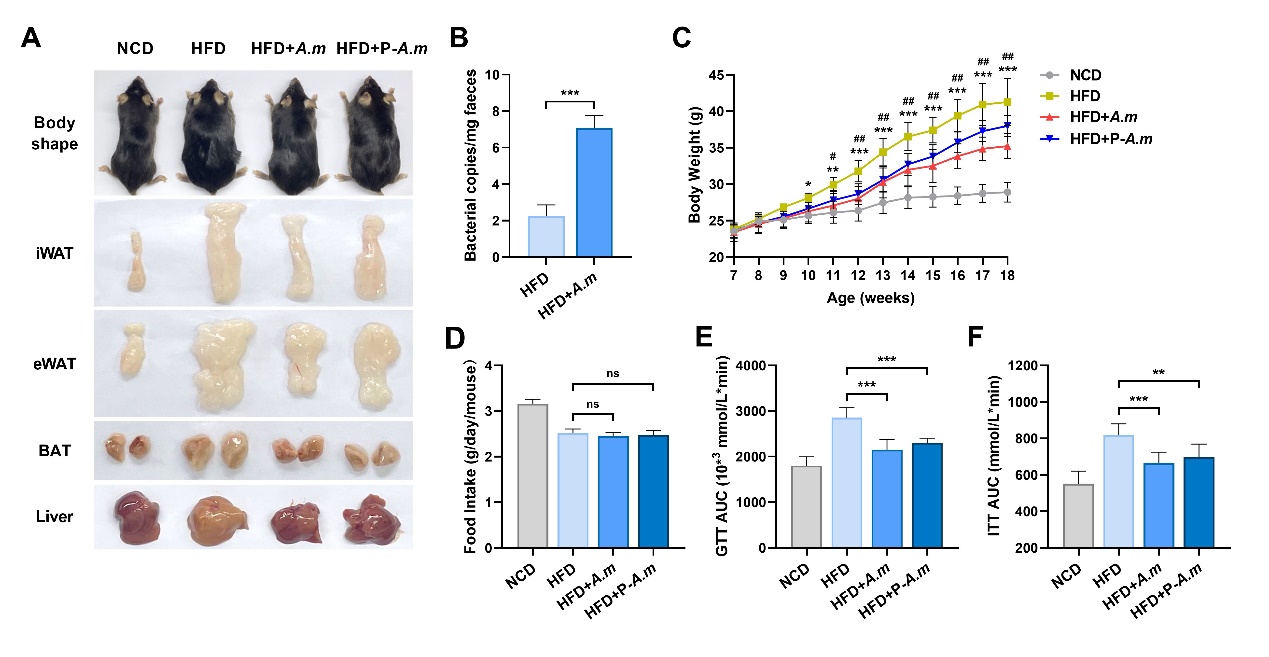


**Supplementary Figure 2. *A. muciniphila* contributes to relieved obesity and metabolic homeostasis.**

**(A)** Representative photographs of mice body shape, iWAT, eWAT, BAT, and liver.

**(B)** Abundance of *A. muciniphila* in mice feces (*n* = 8).

**(C)** Dynamic changes in body weight of mice (*n* = 8). * significant difference between HFD and HFD+*A.m* groups. # significant difference between HFD and HFD+P-*A.m* groups.

**(D)** Food intake of mice.

**(E)** The AUC was calculated based on GTT results (*n* = 8).

**(F)** The AUC was calculated based on ITT results (*n* = 8).

**Supplementary Figure 3**


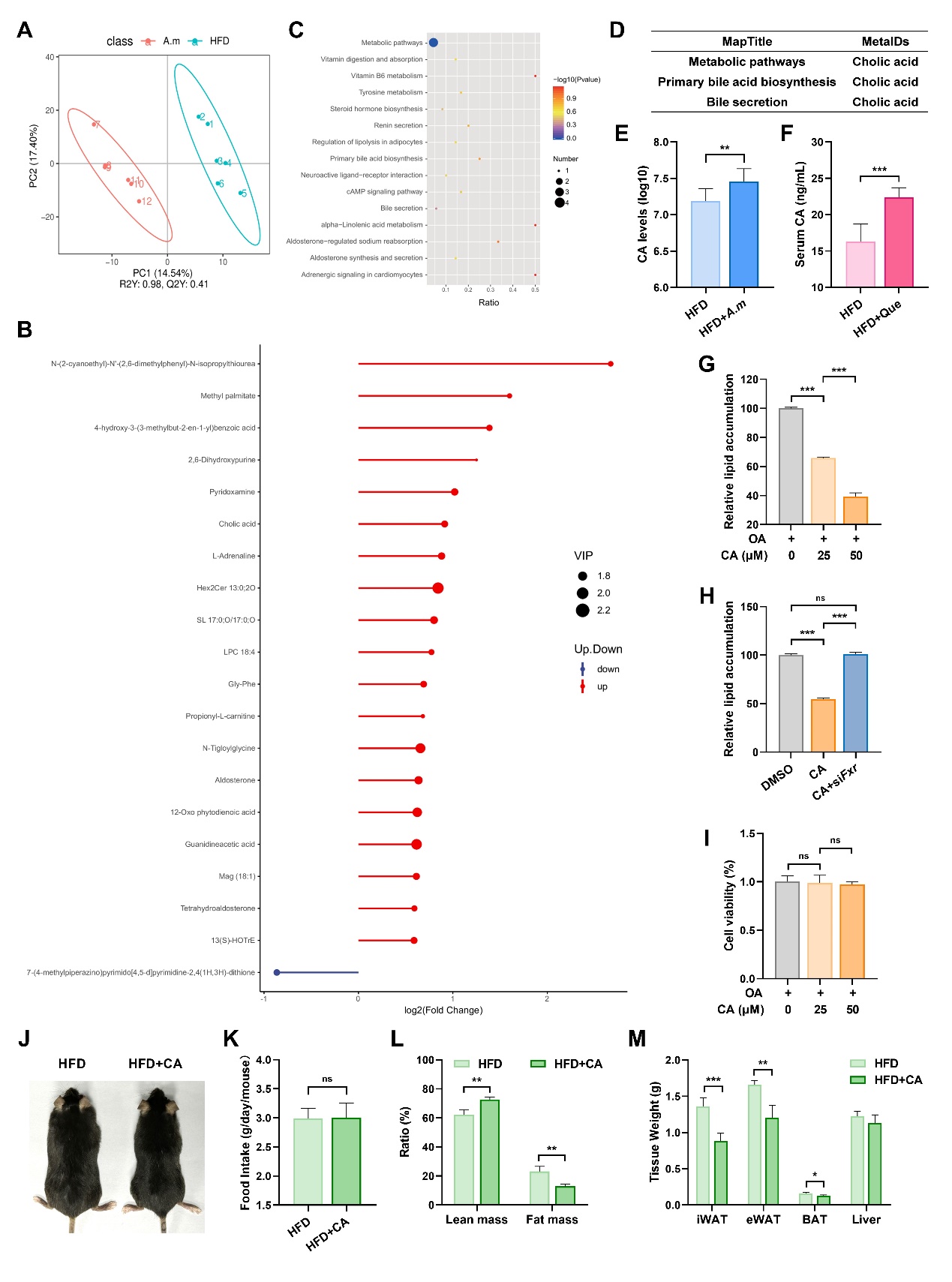


**Supplementary Figure 3. *A. muciniphila*-driven CA confers protection against obesity via FXR signaling.**

**(A)** PLS-DA of metabolite composition between HFD and HFD+*A.m* groups (*n* = 6).

**(B)** Differentially expressed metabolites described in **Fig. 3(A)**.

**(C)** KEGG enrichment analysis of differentially regulated serum metabolites between HFD and HFD+*A. m* groups (*n* = 6).

**(D)** CA is enriched in metabolic pathways, primary bile biosynthesis, and bile secretion.

**(E)** Serum levels of CA in mice between HFD and HFD+*A.m* groups (*n* = 6).

**(F)** Serum levels of CA in mice between HFD and HFD+Que groups (*n* = 6).

**(G)** ORO staining of SVF cells (*n* = 4). Differentiation was induced with CA up to day 6.

**(H)** ORO staining of SVF cells in *Fxr*-silenced or CA-treated groups (*n* = 4).

**(I)** Cell viability of SVF cells after CA treatment at dose of 0 μM, 25 μM, or 50 μM (*n* = 4).

**(J)** Representative photographs of mice body shape.

**(K)** Food intake of mice.

**(L)** Body composition parameters of lean and fat tissues of mice (*n* = 4).

**(M)** iWAT, eWAT, BAT, and Liver weights at termination of study (*n* = 4).

**Supplementary Figure 4**


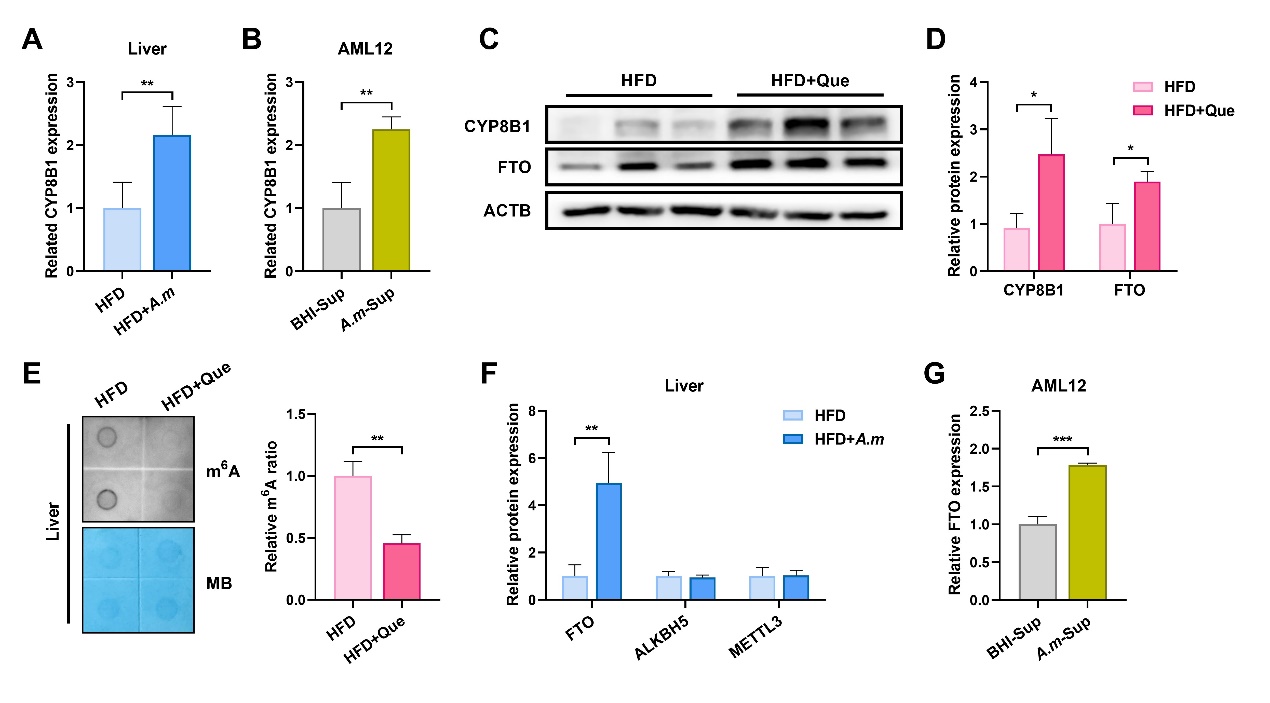


**Supplementary Figure 4. *A. muciniphila* metabolites enhances CYP8B1 expression in an m^6^A-dependent manner to generate more CA.**

**(A)** Protein expression levels of CYP8B1 in livers of mice from HFD and HFD+*A. m* groups (*n* = 4).

**(B)** Protein expression levels of CYP8B1 in AML12 cells treated with BHI-Sup or *A. m*-Sup (*n* = 3).

**(C-D)** Protein expression levels of CYP8B1 in livers of mice from HFD and HFD+Que groups (*n* = 3).

**(E)** mRNA m^6^A modification levels in livers of mice from HFD and HFD+Que groups. Methylene blue staining was used as a loading control (*n* = 3).

**(F)** Protein expression levels of m^6^A regulator in livers of mice from HFD and HFD+*A. m* groups (*n* = 3).

**(G)** Protein expression levels of FTO in AML12 cells treated with BHI-Sup or *A. m*-Sup (*n* = 3).

**Supplementary Figure 5**

**
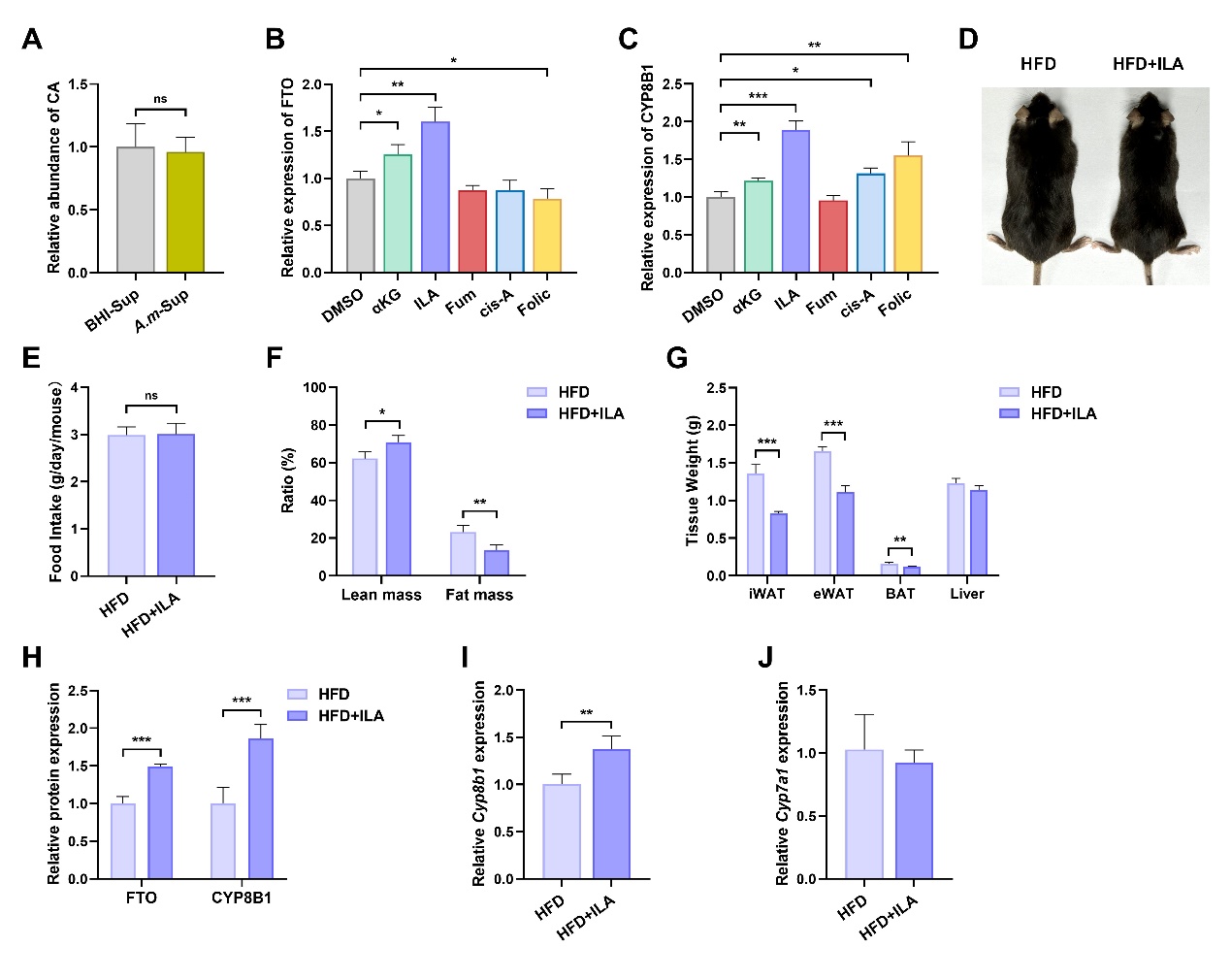
**

**Supplementary Figure 5. *A. muciniphila* metabolite ILA confers to the increased m^6^A-mediated CYP8B1 expression**

**(A)** Relative content of CA between BHI-Sup or *A.m*-Sup (*n* = 3).

**(B-C)** Protein expression levels of FTO **(B)** and CYP8B1 **(C)** in AML12 cells treated with different metabolites (*n* = 3).

**(D)** Representative photographs of mice body shape.

**(E)** Food intake of mice.

**(F)** Body composition parameters of lean and fat tissues of mice (*n* = 4).

**(G)** iWAT, eWAT, BAT, and Liver weights at termination of study (*n* = 4).

**(H)** Protein expression levels of FTO and CYP8B1 in livers of mice from HFD and HFD+ILA groups (*n*=4).

**(I)** mRNA expression levels of *Cyp8b1* in livers of mice (*n* = 4).

**(J)** mRNA expression levels of *Cyp7a1* in livers of mice (*n* = 4).

**Supplementary Table 1. The information of antibodies used in this study.**

| **Antibodies** | **Source** | **Identifier** | **Host** |
| --- | --- | --- | --- |
| ACTB | Huabio | ET1207-67 | Rabbit |
| FTO | Proteintech | 27226-1-AP | Rabbit |
| ALKBH5 | Proteintech | 16837-1-AP | Rabbit |
| METTL3 | Proteintech | 15073-1-AP | Rabbit |
| CYP8B1 | Huabio | ER1907-09 | Rabbit |

**Supplementary Table 2. Sequences of primers used for qPCR.**

| **Gene** | **Forward primer (5’-3’)** | **Reverse primer (3’-5’)** |
| --- | --- | --- |
| *Actb* | GACGGCCAGGTCATCACTATTG | AGGAAGGCTGGAAAAGAGCC |
| *Pparg* | TGGGTGAAACTCTGGGAGATTC | AGAGGTCCACAGAGCTGATTCC |
| *Cebpa* | GGTTTCGGGTCGCTGGATCTCTAG | ACGGCCTGACTCCCTCATCTTAGAC |
| *Fabp4* | GACGACAGGAAGGTGAAGAG | ACATTCCACCACCAGCTTGT |
| *Fxr/Nr1h4* | GGCAGAATCTGGATTTGGAATCG | GCCCAGGTTGGAATAGTAAGACG |
| *Tgr5/Gpbar1* | TGCTTCTTCCTAAGCCTACTACT | CTGATGGTTCCGGCTCCATAG |
| *Cyp8b1* | CTAGGGCCTAAAGGTTCGAGT | GTAGCCGAATAAGCTCAGGAAG |
| *Cyp7a1* | AACAACCTGCCAGTACTAGATAGC | GTGTAGAGTGAAGTCCTCCTTAGC |
| *Fto* | GAGCAGCCTACAACGTGACT | GAAGCTGGACTCGTCCTCAC |
| *Alkbh5* | GCTGTGGTGAGAGAAAGCCT | AGTGGGCAAACACAAGTCCA |
| *Mettl3* | AGTGGCTTTTCATCTTGGCTCTA | GCTGTTTCTTATGGGCCTGGA |
